# Surface Modification of Magnetoactive Elastomers by Laser Micromachining

**DOI:** 10.3390/ma17071550

**Published:** 2024-03-28

**Authors:** Izidor Straus, Gaia Kravanja, Luka Hribar, Raphael Kriegl, Matija Jezeršek, Mikhail Shamonin, Irena Drevensek-Olenik, Gašper Kokot

**Affiliations:** 1 Faculty of Mathematics and Physics, University of Ljubljana, 1000 Ljubljana, Slovenia; izidor.straus@fmf.uni-lj.si (I.S.); gasper.kokot@ijs.si (G.K.); 2 Faculty of Mechanical Engineering, University of Ljubljana, 1000 Ljubljana, Slovenia; gaia.kravanja@fs.uni-lj.si (G.K.); luka.hribar@fs.uni-lj.si (L.H.); matija.jezersek@fs.uni-lj.si (M.J.); 3 East Bavarian Centre for Intelligent Materials (EBACIM), Ostbayerische Technische Hochschule Regensburg, 93053 Regensburg, Germany; raphael.kriegl@oth-regensburg.de (R.K.); mikhail.chamonine@oth-regensburg.de (M.S.); 4 Jožef Stefan Institute, 1000 Ljubljana, Slovenia

**Keywords:** magnetoactive elastomer, MAE, laser micromachining, particle distributions, SEM, EDS

## Abstract

It has been recently demonstrated that laser micromachining of magnetoactive elastomers is a very convenient method for fabricating dynamic surface microstructures with magnetically tunable properties, such as wettability and surface reflectivity. In this study, we investigate the impact of the micromachining process on the fabricated material’s structural properties and its chemical composition. By employing scanning electron microscopy, we investigate changes in size distribution and spatial arrangement of carbonyl iron microparticles dispersed in the polydimethylsiloxane (PDMS) matrix as a function of laser irradiation. Based on the images obtained by a low vacuum secondary electron detector, we analyze modifications of the surface topography. The results show that most profound modifications occur during the low-exposure (8 J/cm^2^) treatment of the surface with the laser beam. Our findings provide important insights for developing theoretical models of functional properties of laser-sculptured microstructures from magnetoactive elastomers.

## 1. Introduction

Magnetic soft materials are composites comprised of magnetic inclusions ranging in size from nanometers to millimeters that are embedded into polymer matrices [1]. One of the categories of such materials that have gained interest in recent years is magnetoactive elastomers (MAEs), sometimes referred to as magnetorheological elastomers [2,3,4,5,6,7,8,9,10,11,12]. Bulk, as well as surface properties of MAEs, can be manipulated with external magnetic fields, which causes restructuring of a magnetic filler in a compliant elastic matrix. This rearrangement of magnetic inclusions induces changes in the elastic moduli [13,14,15,16,17,18,19,20,21] and contributes to magnetostriction [22,23,24,25,26,27,28,29,30,31,32,33,34]. It is also known that an external magnetic field affects such surface properties of MAEs as adhesion [35,36,37,38,39,40], friction [41,42], roughness [39,43,44,45,46], wettability [40,47,48], and optical [49,50].

In 2022, a new method of MAE surface patterning with laser micromachining was introduced by Kravanja et al. [51]. This technique was employed to manufacture magnetically controllable lamellar structures in a surface area of 1 cm^2^ [52,53]. Investigations of the dynamic response of lamellae to the variable magnetic field were carried out, and a simple magneto-mechanical model of the observed phenomena was proposed [52]. Such lamellae can be employed for the transportation of liquid droplets and solid objects [53,54]. Lamellar surfaces also provide control of the droplet impact [55] and switchable wettability [47,52].

However, to date, the effect of laser micromachining on surface topography and composition of MAEs has not been systematically investigated, even though laser ablation of polymers is an established surface processing method [56]. In this work, we provide insight into alternations of MAE surface features caused by laser micromachining, and discuss their implications for further development of theoretical models of magnetically regulated surface properties. We also discuss the importance of the observed modifications for various potential uses of laser-sculptured MAEs.

## 2. Materials and Measurements

**Figure 1:**
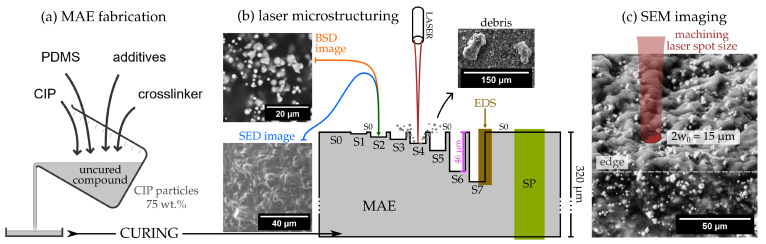
(**a**) Fabrication of MAEs involves several components, mainly a soft polymer and a magnetic filler, in our case, carbonyl iron powder (CIP). The details on the material composition and the fabrication procedure can be found in Supporting Information (SI) and are published elsewhere [47,51]. (**b**) A nanosecond pulsed laser removes parts of the MAE material when focused onto its top surface [51]. Depending on the micromachining parameters, surface layers with different thicknesses are removed. Some MAE debris ejected during the process can remain on the surface. (**c**) The processed surface parts (samples) are analyzed with scanning electron microscopy (SEM). Different sample-detector combinations are annotated with different colors: S0–S7 were measured with a BSD (orange) and SED (blue), SP with a BSD (green), and energy-dispersive X-ray spectroscopy (EDS) measurements are represented in brown. Additionally, the size of the laser spot during the micromachining process is plotted in scale.

Our investigation involved eight samples fabricated from a common MAE film with an approximate thickness of 400 μm. The material, synthesized via crosslinking of a mixture of uncured polydimethylsiloxane (PDMS) and 75 wt% of carbonyl iron powder (CIP), had a shear storage modulus of $G^{\prime }\approx 15$ kPa in the absence of a magnetic field [47]. Samples underwent micromachining treatments with variable laser power and number of beam passages to assess surface modifications caused by different structuring parameters, as illustrated in Figure 1.

Laser micromachining was carried out utilizing a nanosecond pulsed Nd:YAG fiber laser with an average power of 20 W and a repetition frequency of 30 kHz. The scanning velocity of the beam was 500 mm/s. The peak power of laser pulses was around 7 kW [51]. The exposure energy of the MAE surface was estimated from the pulse optical energy and laser beam diameter $2w_{0}=15$ μm (defined as the width at which the power per unit area is a factor of 1/e lower than the peak irradiance). We took into account only one pulse-per-region impact due to large laser translation (≈16.7 μm) during pulses, and therefore we did not overlap them. The distance between parallel laser line passages was also chosen to be equal to $2w_{0}$ in order to avoid overlap. It should be noted that the crater size resulting from a single high-energy laser pulse is larger (≈25 μm) than the laser beam diameter (Figure 1c), because the intensity profile has a bell-like shape. This means that even though we did not overlap the laser pulses according to the parameter $w_{0}$ we did overlap the affected area by ≈50% [47].

The variations among the samples stemmed from different fractions of the full laser power used for processing (20%, 40%, 60%, 80%, 100%) and the number of passages (1, 2, 4) made across the same sample, each one removing a few μm thick layer of material. An overview of the structuring parameters used for the preparation of the samples named S0–S7 is presented in Table 1. All samples were fabricated side by side on the surface of the same large film of MAE, therefore excluding possible fabrication variations between different batches. Seven samples processed with different laser exposures were labeled as S1–S7, while S0 denoted a pristine (unprocessed) surface. The investigation also involved an additional sample that was cut with a scalpel across its thickness and observed from the side. This specimen was denoted as the SP (side profile) sample. The spatial arrangement of the samples is depicted in Figure 1b. The thickness of a layer removed at specific micromachining parameters was estimated from optical microscopy imaging with a selective focus at sample borders.

Scanning electron microscopy (SEM), with its multiple detection options, is a very suitable tool for assessing different structural and compositional properties of composite materials, such as MAEs. We used two SEM instruments (Thermo Scientific™ Axia™ ChemiSEM™ Scanning Electron Microscope (Waltham, MA, USA) and Keithley Instruments Inc. (Solon, OH, USA) Helios Nanolab 650) equipped with a concentric backscatter detector (BSD), secondary electron low vacuum detector (SED), and an energy-dispersive X-ray spectrometer (EDS). Most measurements were performed on Axia™ ChemiSEM™ and a few images were taken with the Helios Nanolab 650 (Figure 1c).

The BSD is used to resolve constituents with a different atomic number (Z-number), in our case between iron and other non-metals present in the polymer matrix. A high acceleration voltage (30 kV) was used to obtain an electron penetration depth of ≈5 μm [57]. Conversely, the SED was operated in conjunction with a low acceleration voltage of 2 kV, enabling the imaging of only the topmost layer of the surface (≈10 nm). Low vacuum was needed to avoid drifting, as imaging at low accelerating voltages produces less signal per dwell time. The resulting topographic contrast stems from the inclination between a specific surface region and the electron beam, impinging perpendicular to the sample plane. Steeper local inclinations generate higher secondary electron emission and appear brighter in the SED images. The EDS detector was used for comparison of elemental composition between the samples. For details on SEM procedures, see Supplementary Materials.

A few nanometers’ thick layer of carbon was sprayed over the samples to prevent the build-up of electric charge. The samples were glued on pin holders with carbon tape, providing additional charge dissipation. The sample fabrication, structuring, and imaging process are sketched in Figure 1, where (a) sketches the fabrication procedure, and (b) and (c) the micromachining and imaging processes. The depths of samples S1–S7 with respect to sample S0 are shown to scale; however, their width and the entire MAE thickness (320 μm) are not.

## 3. Results

### 3.1. Cross-Sectional Profile Analysis

**Figure 2:**
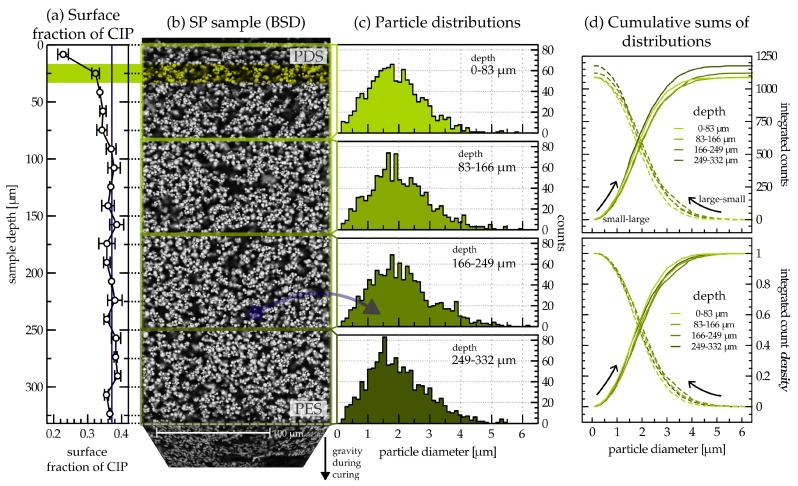
(**a**) Surface fraction of particles along the depth of the SP sample. Each point is extracted from the corresponding slice (an example is highlighted with a thick green ribbon) of image (**b**), which shows the whole SP. A BSD reveals high Z-number atoms; therefore, carbonyl iron powder (CIP) particles are shown in white. The sample is annotated with PES (particle-enriched side) and PDS (particle-depleted side) labels. It is divided into four slices from which the particle diameter distributions shown in (**c**) are extracted. Each slice is >80 μm thick. (**d**) Cumulative sums of particle distributions from 0 to 6 μm in diameter. The top graph shows the absolute number of counts for the integrated bins, while the bottom one shows the count density normalized to 1. The direction of the summation is marked with arrows for the small-to-large (solid lines) and large-to-small (dashed lines) diameters.

Polymer curing takes some time, and therefore the density difference between the filler (in our case, iron) and the polymer matrix (in our case, PDMS) can lead to sedimentation. In order to gain insight into how sedimentation may alter the homogeneity of the investigated MAE, the SP (side profile) sample was inspected along its entire cross-section by using a BSD with high accelerating voltages. Multiple images were captured and joined into a panoramic image. A custom-written ImageJ macro was used to extract iron particle diameter distribution, separating closely packed particles while assuming spherical shapes. Figure 2 shows the quantitative analysis outcomes, presenting four particle diameter distributions extracted from distinct slices along the thickness. In terms of sample orientation during curing, the brightest slice represents the uppermost layer, while the darkest one corresponds to the bottom layer.

Figure 2a reveals a decrease in particle surface fraction at the particle-depleted side (PDS), i.e., at the MAE film top surface. It was obtained by masking all particles at a corresponding depth and calculating the masked surface area fraction. The particle-enriched side (PES), i.e., the bottom side of the film, does not exhibit a distinguishable increase in particle surface fraction, which is in agreement with previous published work [47], where we also adopted the PES and PDS notation [39,47]. The cumulative sums of the particle counts and count densities are presented for each of the four slices, integrating particles from small to large diameters and, inversely, from large to small diameters (Figure 2d). We present both the absolute (Figure 2d top) and the normalized (Figure 2d bottom) cumulative sum. The latter makes it possible to compare the distributions in terms of the relative share between small and large particles. In the direction of summation from small to large diameters (Figure 2d bottom, solid lines), we observe that the cumulative sum for the smallest depth is larger than the rest before reaching 1.

### 3.2. Particle Size Distribution

Samples S0–S7 were imaged under the same parameters as in Section 3.1. Each sample underwent analysis at four different locations performed with BSD imaging, capturing particles in the top layers of the material.

**Figure 3:**
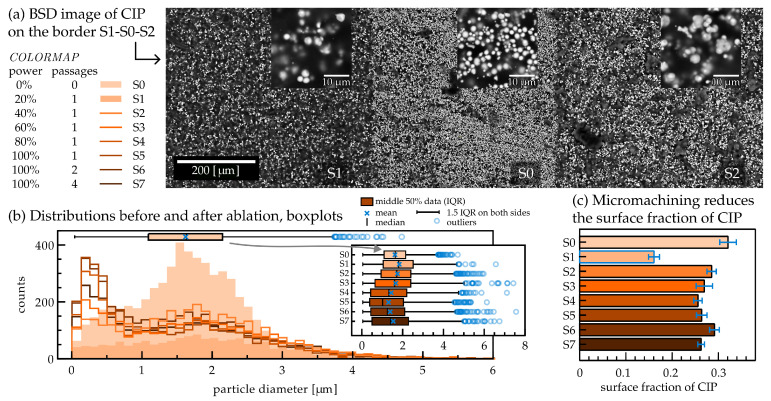
(**a**) A BSD image of parts of the samples S1 and S2 with a region of unstructured material (sample S0) in between (images of other samples are presented in Supplementary Materials Section “Raw data”). CIP particles are shown in white, showcasing a visible change in their surface fraction amongst the samples. The inserts are parts of the magnified regions from which the particle diameter distributions were extracted. (**b**) Particle diameter distributions as obtained from four equally sized regions of every sample. Above the histograms lies a boxplot representation of the distribution for sample S0, and the other boxplots are shown in the inset. IQR stands for the interquartile range where 25–75% of particles are found. (**c**) Analogous to Figure 2a, the surface fraction of CIP particles is shown for all samples S0–S7.

Figure 3a provides a qualitative comparison between micromachined and pristine surfaces, as it shows a part of the sample that contains regions of S1 and S2 separated by a slice of the unprocessed material, S0. Figure 3b displays the particle diameter distributions extracted from magnified regions of samples S0–S7. A notable difference in distribution shape appears between the non-treated sample, S0, and the treated samples. Specifically, the number of smaller particles (diameter < 1 μm) increases in samples S2–S7. Additionally, some larger particles appear after the micromachining process, as the outliers in the adjacent boxplot show. Size distributions obtained for treated surfaces S1–S7 do not show any significant statistical differences, if we exclude the smaller particles (diameter < 1 μm). However, they differ statistically from the pristine sample, S0, as confirmed by the Kruskal–Wallis statistical test (see Supplementary Materials Section S3).

The analysis of the particle area fraction (Figure 3c) reveals that the sample irradiated by the lowest laser power (20%, S1) stands out from the rest, exhibiting a minimal value of particle surface fraction below 0.2. This feature can be readily noticed in (a), where the weakly micromachined part of the sample (S1) appears darker than the pristine sample (S0) or 40% power-treated sample (S2).

### 3.3. Qualitative Assessment of Surface Topography

**Figure 4:**
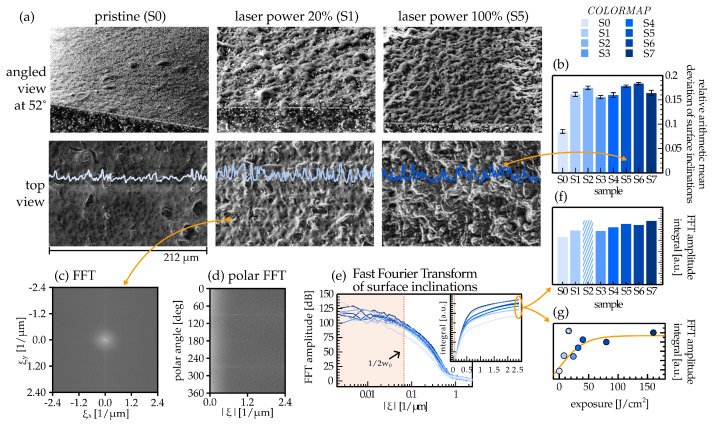
(**a**) Secondary electron images of three different samples taken at the angle of 52° with respect to the surface normal and images of their top view. Top-view images are fitted with exemplary intensity profile plots highlighting surface inclinations. The gray dashed lines are used to mark the edge between the top surface and the cross-cut area. Images of other samples are presented in Supplementary Materials Section “Raw data”. (**b**) Measurements of relative arithmetic mean deviation of surface inclinations averaged over a larger 2D surface for each sample. (**c**) FFT of the top-view images deconstructed into a polar plot in spatial frequency space in (**d**). (**e**) FFT amplitudes averaged over all polar angles for each sample. A shaded region represents spatial variations larger than the laser’s spot size. The amplitudes to the right of the dotted red line are integrated over the remaining spatial frequencies and shown in the inset. Total integrated values of FFT amplitudes are shown in (**f**,**g**), with the x-axis depicting sample names and laser exposures used in sample fabrication, respectively.

The investigation into surface topography modification was conducted by the SED imaging, examining the ≈10 nm thick topmost surface layer of the samples. Figure 4a shows the side and top profiles of selected samples imaged at lower magnification. Initial images underwent background subtraction using a rolling ball method to level them on a common plane. Images were obtained by a Helios Nanolab 650 device and subjected to contrast limited adaptive histogram equalization (CLAHE) to mitigate local contrast adjustment.

Due to the absence of an absolute height scale, the subsequent analysis of mean deviations of surface inclinations (Figure 4b) was confined to a binary classification, which distinguished between pristine and treated samples. A parallel fast Fourier transform (FFT) (c,d) of the images was performed to categorize the samples based on their micromachining parameters. The sample S0 exhibited the lowest amplitudes at spatial frequencies ξ above the reference frequency given by the inverse laser beam diameter (red dashed line). Figure 4e illustrates the results obtained by integrating the amplitudes of the FFT signals over the polar angle in ξ-space, effectively leading to the results shown in (f). Figure 4g shows the same results as a function of the exposure energy.

### 3.4. Surface Chemical Composition

**Figure 5:**
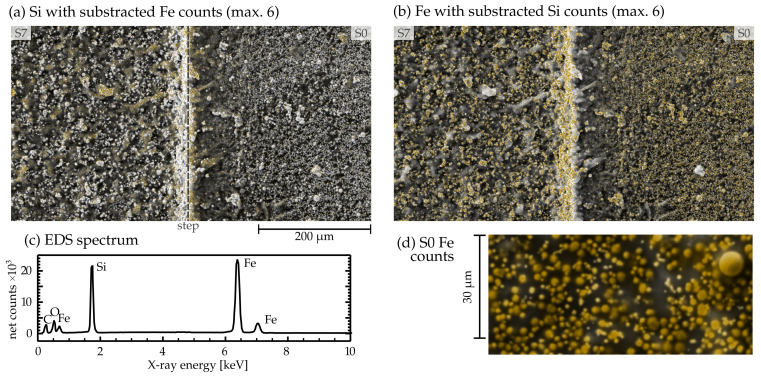
EDS count map overlaid over an SEM BSD image of the border between S7 and S0 (see Figure 1b, for EDS images and spectra of all samples see Supplementary Materials Section “Raw data”). Characteristic X-ray photons originate mostly from iron and silicon, with less signal coming from oxygen and carbon, the latter of which was used for coating the samples. The images highlight parts of the MAE surface with prevailing silicon (**a**) or iron atoms (**b**), as obtained by subtraction. (**c**) The EDS spectrum of the regions shown in (**a**,**b**). (**d**) Close-up of iron particles in S0 with colored EDS Fe counts.

EDS mapping provides information on the elemental composition of the surface inspected by the SEM imaging. We chose a part of the MAE containing the boundary between S7 and S0 (see Figure 1b) to discern possible changes in chemical composition between the two. Count maps for different elements are overlaid over a BSD image, and the corresponding spectrum is shown in Figure 5.

The surface of both laser-treated and pristine MAE comprises mostly iron and silicon. Figure 5a,b highlights parts of the surface with prevailing silicon and iron counts, respectively. We observed that micromachining creates or uncovers large 10–20 μm portions of material not containing any iron particles.

## 4. Discussion

Traditional optical microscopy is not very effective for imaging MAE surface structures due to the black color appearance of the material and its specific affinity for reflecting only those light rays incident upon the iron particles. In combination with the transparency of the PDMS matrix, this property makes a distinction between the actual surface and the underneath surface layers quite difficult, particularly in the interferometric optical surface imaging methods. In addition to its intrinsic submicrometer resolution, the advantage of the SEM technique is that, by using different detectors and imaging parameters, one can tune the depth of focus so that surface layers of different depths can be analyzed.

The analysis of the cross-section of the MAE film highlights a decrease in particle concentration at the particle-depleted side (PDS) at the top in comparison with the rest of the sample, a phenomenon already documented in the literature [39,43,47]. The effect is attributed to sedimentation of CIP particles during the curing process. This has to be taken into account when modeling surface phenomena using bulk properties. If a uniform particle density along the thickness is desired for an application, a particle-depleted layer can be removed by laser micromachining—for our material composition this means approximately an 80 μm thick layer of the material. The presence of the PES is considered to be an important feature of our material that facilitates reversible changes in surface roughness upon application of a magnetic field. This leads to significant changes in the surface roughness that profoundly effect adhesion, wettability, and optical properties. Observing the normalized cumulative sums of count densities (Figure 2d bottom, solid lines), we found an indication that the particle distribution on the PDS side has a larger share of smaller particles than other depths, which should be investigated in greater detail and with larger statistics in a future work.

The prominent presence of very small particles in the size distributions of samples S2–S7 (Figure 3b) is ascribed to the generation of fine iron particles during the micromachining treatment, resulting from the destruction of CIP particles [58]. Additionally, the emergence of larger conglomerates, seen as outliers in the boxplot, is probably a consequence of CIP particles melting and coalescing together [59]. Through these two processes, the size distribution of particles at the surface undergoes significant changes that need to be considered when modeling surface properties.

The observed dip in particle surface density for sample S1 in Figure 3c is quite intriguing. It is crucial to remember that all samples were derived from the same MAE film, micromachined side by side on a pristine surface. Therefore, if the surface of S1 were treated with a larger laser exposure, for instance, 16 J/cm^2^, the resulting surface would resemble sample S2.

The lower particle surface density of S1 stems from a lower particle-to-polymer ratio. Considering the relatively constant particle density within the small depth range of micromachining, the increased presence of polymer obscures the detection of particles underneath. This thicker layer of polymer at the top surface is unique to the S1 sample and is likely linked to the effects of the laser treatment. The absorption of near-infrared light produced by our laser in PDMS is negligible compared with iron. [60,61] This means effectively that all the delivered light is absorbed by the particles. As our working hypothesis, we propose that exposures below 10 J/cm^2^ heat the densely CIP-populated pristine surface sufficiently for the iron particles to evaporate from the surface while the surrounding polymeric material remains on it. Additionally, we know that low laser fluence correlates approximately linearly with low ablation depths [62,63,64], indicating that laser pulses do not affect the particles in the layers under the polymer surface. The limited depth of processing hence creates a unique scenario with selective removal of only one component (iron) of the composite medium. The remaining polymer-rich surface then obstructs the imaging of iron particles underneath, resulting in an effective lower surface fraction.

Once the laser exposure is sufficiently large (case of samples S2–S7), the heated iron particles also induce removal of the polymeric material, so both components are affected, despite the low absorption of PDMS to near infra-red light. Consequently, the surface of the MAE maintains a relatively constant particle density and produces craters in PDMS larger than the laser spot size, regardless of exposure. The micromachining process in our experiments was performed on the particle-enriched side (PES) of the MAE film.

Secondary electrons induced by low energy primary electrons originate only from a surface layer much thinner than the average size of CIP particles (≈10 nm, see Supplementary Materials Section S2) [57]. Their number coefficient (how many reach the detector) and, consequently, the image brightness, increases with the increasing inclination of the surface with respect to the primary electron beam. Analysis of the relative arithmetic mean deviation of this signal (Figure 4b) provides insight into the density and size of surface inclinations. A larger number indicates greater deviations in surface inclination, while a lower one denotes a more leveled surface. As images were captured at different brightnesses and contrasts, histogram equalization was necessary for comparison, albeit at the cost of diminished information about the absolute scale of surface inclinations. To enhance binary classification, the change in surface topography was further validated with frequency analysis (Figure 4c–g), indicating an increase in inclination frequencies on length scales smaller than or comparable to the laser beam diameter. Variations on the wavelengths larger than the beam diameter were not included in the analysis, as a single-line laser passage could not have created them and they are likely the result of multiple adjacent laser passages. The amplitude of small-scale spatial variations monotonically increases with the exposure energy of the samples, except for sample S2. The apparent low-ξ surface inclinations of S2 result from overexposure (pixel saturation) during SEM imaging, translating into more pronounced low-frequency amplitudes.

Knowing the surface chemical composition could be important for theoretical considerations of the interaction between the laser-micromachined MAE surface and different objects, e.g., water droplets or glass spheres. Removed particles typically produce grooves of removed material, so CIP is usually not found in the topmost layer of the MAE, as shown in Figure 4a. Consequently, an object making contact with the surface of a micromachined MAE primarily interacts with the polymer, not the polymer–particle composite. Modeling the magnetically tunable surface contact properties of the MAE could be simplified by considering only the distortion of the surface topography of a polymer layer due to iron particle reconfiguration underneath and disregarding their interactions with the (non-magnetic) object in contact.

For a more quantitative analysis of the surface roughness of laser-processed MAEs, atomic force microscopy (AFM) imaging could be performed. Our material is very soft ($G^{\prime }\approx 15$ kPa) and somewhat sticky, and therefore AFM measurements should be done with great care in either the “tapping mode” or the “non-contact mode”. The spatial resolution of the probe should be optimized by employing a suitably sized tip. Alternatively, the images acquired by SEM could be scaled by incorporating an object of known size into the MAE surface.

As characteristic X-rays originate from multiple layers beneath the surface, the EDS measurement in Figure 5 is superimposed over the BSD-acquired image. Examining the elemental composition captured with EDS assures that the iron particles retain their spherical shape after laser treatment, notwithstanding the formation of some clumps. Furthermore, it is evident that laser irradiation causes the creation of larger portions of polymer-exclusive material, as previously established.

## 5. Conclusions

In this study, the structure of pristine and laser-micromachined MAE composites was investigated using several detectors of a scanning electron microscope. Side profile images identified that the particle-depleted layer was about 80 μm thick, and should be adequately considered when modeling such material. We have demonstrated that this layer can be removed by laser micromachining but at the expense of increased surface roughness. Low-exposure (8 J/cm^2^) micromachining can produce a much lower particle surface density and affects roughness less than higher exposures. More detailed investigation into the low-exposure micromachining regime is required to determine the possibility of tuning the iron particle surface density.

MAE surface topography was elucidated by analysis of surface inclinations, demonstrating that higher laser energy exposure produces surfaces with more frequent or more pronounced inclinations. We found this effect to saturate after one or two laser passages with high enough exposure (>40 J/cm^2^)—important information for any technological use of laser micromachining of MAE, where repeatable surface roughness is a key parameter. Further possible improvements in the methodology are discussed, because absolute roughness determination cannot be performed with the presented analysis.

Finally, elemental analysis showed that after micromachining larger areas emerge where there is only polymer present, and these populate the highest points of a structured MAE. This feature should be considered when modeling surface properties of MAEs because it indicates that objects placed on MAEs will be primarily in contact with the polymer and not the magnetic particles.

## Figures and Tables

**Table 1:** Sample structuring parameters.

Sample	Average Laser	Peak	Num. of	Exposure	Removed Layer
Label	Power [%]	Power [kW]	Beam Passages	[J/cm^2^]	Thickness [μm]
S0	0	0.0	0	0	0
S1	20	1.4	1	8	2 ± 1
S2	40	2.8	1	16	6 ± 2
S3	60	4.2	1	24	9 ± 2
S4	80	5.6	1	32	14 ± 2
S5	100	7.0	1	40	23 ± 3
S6	100	7.0	2	80	49 ± 3
S7	100	7.0	4	160	62 ± 3
SP	0	0.0	0	0	/

## Data Availability

The raw data supporting the conclusions of this article will be made available by the authors on request.

## Supplementary Materials

The following supporting information can be downloaded at: https://www.mdpi.com/article/10.3390/ma17071550/s1, Supporting Information includes CIP dust distributions (Figure S1), SEM penetration depth estimates, Kruskal–Wallis statistical comparison of particle diameter distributions (Figures S2–S4), and unprocessed BSD, SED (Figure S5), and EDS images (Figures S6 and S7), along with EDS spectra (Figure S8).
