# Supplementary material for: Surface Modification of Magnetoactive Elastomers by Laser Micromachining
Source: Materials (Basel). 2024 Mar 28;17(7):1550. doi: 10.3390/ma17071550 (PMC11012975; doi:10.3390/ma17071550)
Supplement: Supplementary file 1 [file materials-17-01550-s001.zip › materials-2916474-supplementary.pdf]

Surface modification of magnetoactive elastomers by laser micromachining: Supporting Information

Izidor Straus [1], Gaia Kravanja[2], Luka Hribar[2], Raphael Kriegl[3], Matija Jezeršek[2], Mikhail Shamonine[3], Irena Drevensek-Olenik[1,4,*] and Gašper Kokot[1,4]

March 28, 2024

* Correspondence: irena.drevensek@ijs.si
[1] Faculty of Mathematics and Physics, University of Ljubljana; izidor.straus@fmf.uni-lj.si
[2] Faculty of Mechanical Engineering, University of Ljubljana; gaia.kravanja@fs.uni-lj.si, luka.hribar@fs.uni-lj.si, matija.jezersek@fs.uni-lj.si
[3] East Bavarian Centre for Intelligent Materials (EBACIM), Ostbayerische Technische Hochschule Regensburg; raphael.kriegl@oth-regensburg.de, mikhail.chamonine@oth-regensburg.de
[4] Jožef Stefan Institute; gasper.kokot@ijs.si

S1 MAE composition

The base polymer VS 100000 (vinyl-functional polydimethylsiloxane) for addition-curing silicones, the chain extender Modifier 7 15 (SiH-terminated p olydimethylsiloxane), t he r eactive d iluent p olymer MV 2000 (monovinyl functional polydimethylsiloxane), the crosslinker 210 (dimethyl siloxane-methyl hydrogen siloxane copolymer), the Pt-catalyst 510 and the inhibitor DVS (vinyl-tetramethyldisiloxane) were provided by Evonik Operations GmbH, Specialty Additives, Geesthacht, Germany. The silicone oil WACKER® AK 10 (linear, non-reactive polydimethylsiloxane) was purchased from Wacker Chemie AG, Burghausen, Germany. The carbonyl iron powder (CIP, type SQ) was used as the ferromagnetic filling. It was provided by BASF SE Carbonyl Iron Powder and Metal Systems, Ludwigshafen, Germany,

Table 1: Composition of MAE used in our experiments. Numbers given are in mass concentration percents (wt%).

CIP	VS 100,000	MV 2000	AK10	Modifier	CL 210	Inhibitor	Catalyst
74.79	7.07	1.26	16.62	0.03	0.12	0.03	0.08

S1.1 CIP dust distribution

Fig. S1 shows the distribution of particle diameters of the carbonyl iron powder and compares it to the distribution of the CIP embedded into MAE. The distributions are qualitatively different.

S2 Scanning Electron Microscopy (SEM)

Because the elemental analysis is calculated based on the characteristic X-ray energies of atoms positioned in a large fraction of the electron interaction volume [1], the spatial resolution of EDS measurement was reduced. Due to hardware limitations, the measurement took place in high vacuum, where the reference image was prone to drifting. To mitigate this, a low dwell time was employed and to increase signal strength a high accelerating voltage was needed.

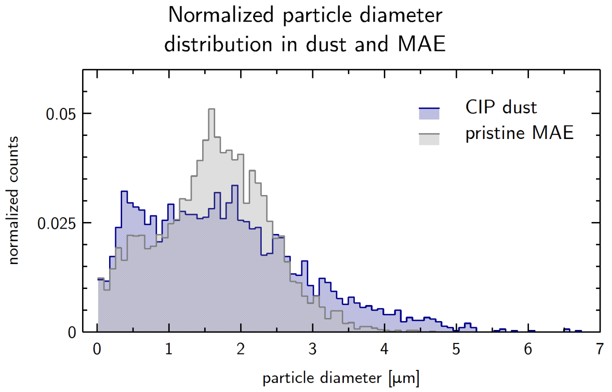

Figure S1: Normalized histograms of particle diameters in pure CIP dust and the pristine MAE (S0).

S2.1 Electron penetration depth estimation

During SEM imaging, electrons are accelerated towards the studied material, producing a complex system of different particles and excitations. In our study we were measuring backscattered electrons and secondary electrons.

First we estimate the depth/size of the interaction volume for MAE using a Kanaya-Okayama range equation [1]:

$$R_{K-O}(\mathrm{nm}) = 27.6(A/Z^{0.89}\rho)E_0^{1.67}, \tag{1}$$

where where A is the atomic weight (g/mol), Z is the atomic number, ρ is the density (g/cm^3), and E_0 is the incident beam energy (keV).

For MAE calculations we assume the following:

1. We consider only the polymer matrix without the particles. We assume that, if an electron hits an iron particle, it stops penetrating the material. This is partly true, as the penetration depth for pure Fe for 30 keV electrons is around 3.2 µm, which is just a little larger than our mean particle diameter.
2. The largest atom in the polydimethylsiloxane (PDMS) is the silicon atom, so we calculate with A_{Si} and Z_{Si}.
3. For the material density we take the PDMS density as the prevailing component in the polymer matrix.

Given our assumptions, the interaction depth of electrons with 30 keV is about 21 µm. However, the backscattered and secondary electrons can only originate from a top part of this interaction volume.

S2.1.1 Origin of backscattered electrons

Interpolating from Table 2.2 in Chapter 2.2 from [1], the backscatter electrons penetration depth ratio for silicon is approximately $D/R_{O-K} = 0.247$, for MAE probably a bit more. Still, using this number we can calculate that the SEM backscattered electrons originate from the top few µm of the surface:

$$D_{\mathrm{BSE}} \approx 5\ \mu\mathrm{m}. \tag{2}$$

When looking at the CBS images we are therefore examining the top 5 µm of the MAE surface.

S2.1.2 Origin of secondary electrons

Secondary electrons are generated throughout the interaction volume, but only few manage to escape to material for us to detect. The mean escape depth can be estimated from the Kanaya-Ono equation [1]:

$$D_{\mathrm{SE}}(\mathrm{nm}) = 0.267AI/(\rho Z^{0.66}), \tag{3}$$

where I is the first ionization potential (eV). Considering A_{Si}, I_{Si}, Z_{Si} and ρ_{PDMS}, the escape depth for secondary electrons is estimated to be:

$$D_{\mathrm{SE}} \approx 11\ \mathrm{nm}. \tag{4}$$

When imaging the surface with a LVD, we are therefore analyzing information originating from the first 11 nm of the surface. To increase secondary electron yield we need to employ lower accelerating voltages [1], and 2 keV was the lowest at which we could have obtained images.

S3 Kruskal-Wallis statistical test for comparing distributions

To compare the distributions of the side slices of SP and the top views of S0–S7 a Kruskal-Wallis statistical test is employed, which is useful for comparing distributions of different size.

The null hypothesis is always that the distributions are the same. The statistical test returns a p value for comparing all distributions for all possible combinations (eg. S0 with S1, S0 with S1 and with S2, and so on). We choose a $p = 0.05$ threshold. If the calculated p value is larger than our threshold, we cannot reject the null hypothesis and therefore cannot claim, that the distributions are statistically different.

S3.1 Side profile slices

The similar distributions in the SP are plotted on Fig. S2 with 1 denoting the PDS and 4 the PES. The most similar distributions come from the middle slices of the SP, in this notation the slices 2 and 3. Interestingly, the distributions for 1 and 4 also appear very similar. All distributions are directly or indirectly similar between themselves as illustrated in the nodes graph on Fig. S4.

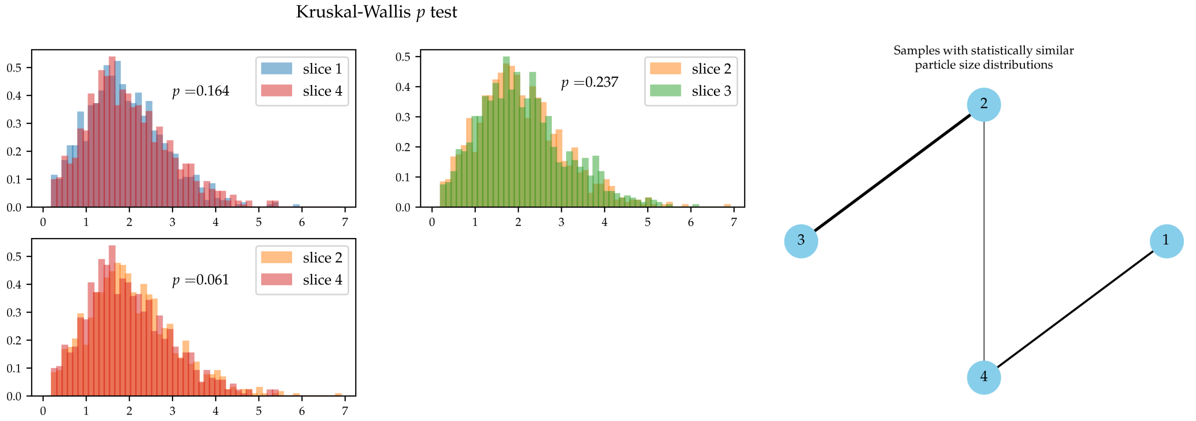

Figure S2: Similar SP distributions overlaid over each other with calculated p value from the Kruskal-Wallis statistical test. Connections between similar distributions are plotted in the node graph with thicker line connecting more similar distributions (the thickness corresponds to the calculated p value).

S3.2 Distributions after ablation

Similarly to the SP, the top view S0–S7 were also compared for similarities. Since ablation increases the number of smaller < 1 µm particles, those were not considered in the comparison, as they effect the distributions too much. Fig. S3 shows the similar distributions. S0 is not found anywhere, meaning it is statistically different from the others.

S1–S7 however are all directly or indirectly similar between themselves as illustrated in the nodes graph on Fig. S4.

S4 Raw data

In the following subsections we present raw SEM images from which the analysis in the main text was conducted.

S4.1 BSD images for particle distributions

A backscatter detector (BSD) was used to gather particle diameter distributions for samples S0-S7. Each image was taken at ×2500 magnification and with a 30 keV acceleration voltage. Samples ere imaged at

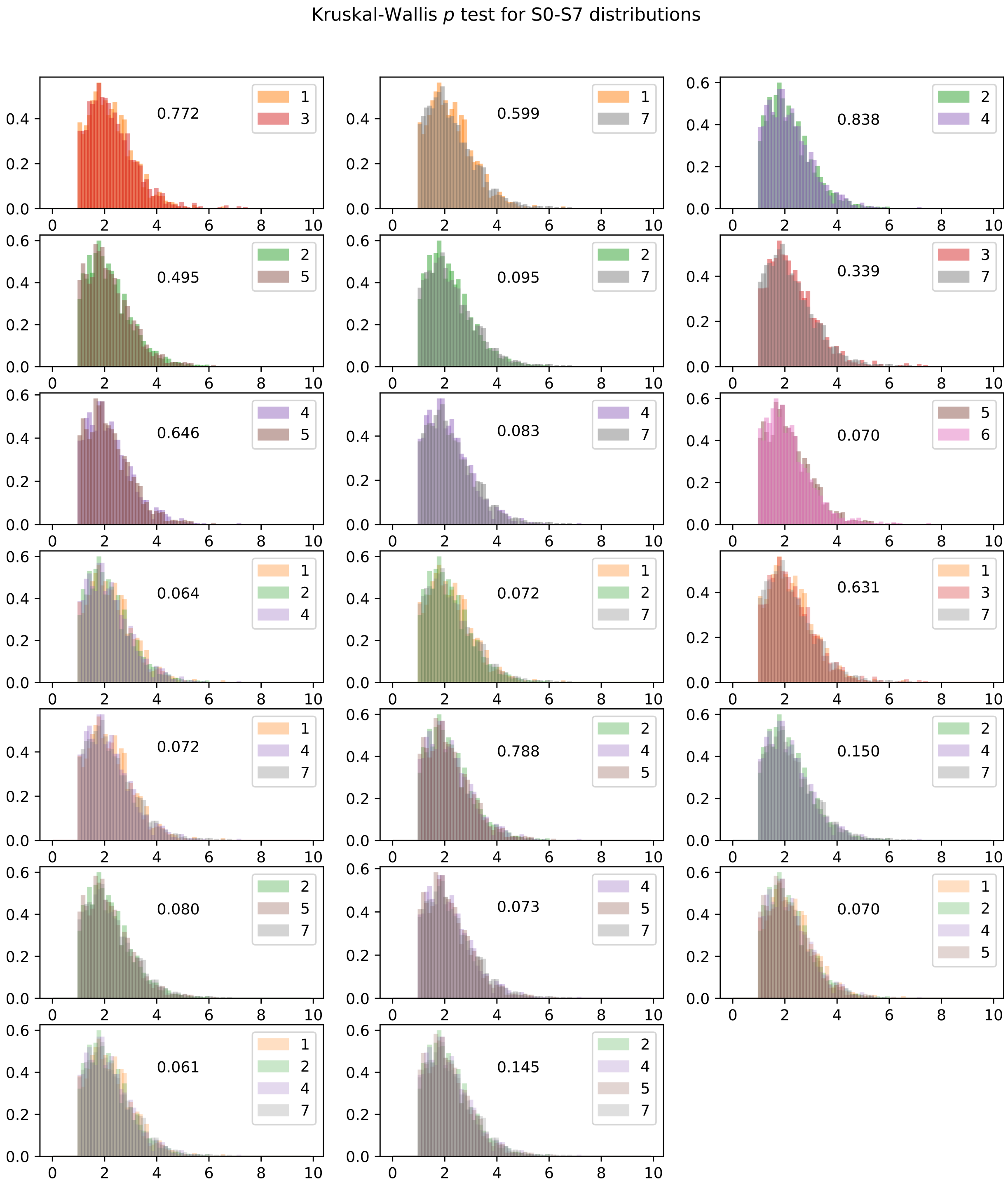

Figure S3: Similar distributions after ablation overlaid over each other with calculated p value from the Kruskal-Wallis statistical test.

4 different r andom l ocations i n o rder t o i ncrease t he n umber o f c ounts t he d istributions, m aking them more statistically relevant (see Fig. 3 (b) in the main text). A custom ImageJ macro was employed to extract particles from the images.

S4.2 SED images for surface topography

A secondary electron detector (SED) was used to asses the surface topography of samples S0–S7. Images, presented in Fig. S5, were taken in low vacuum conditions (0.7 mbar), at a lower magnification ($\times650$) and at low acceleration voltage (2 keV).

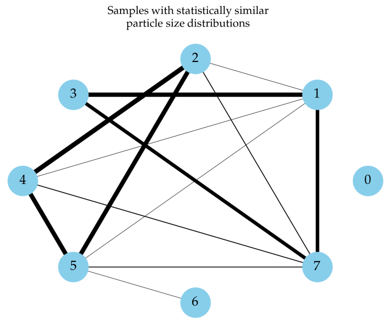

Figure S4: Connections between similar distributions are plotted in the node graph with thicker lines connecting more similar distributions (larger p value). S0 is dissimilar to all the others.

In the FFT-based topography analysis, described in the main text, the S2 sample is shown as an outlier on the FFT amplitude integral vs. exposure plot (Fig. 4 (g)). It exhibits some relatively large overexposed areas. These overexposures are a common consequence of SEM imaging due to the build up of electric charge or incorrect detector sensitivity settings. Larger surfaces of overexposed pixels contribute strongly to the high spatial frequency amplitudes in the FFT analysis, rendering overexposed measurements unfit to compare with other measurements. Additionally, we should note that the FFT analysis is only valid when all the measurements are performed in the same session and on the same sample, due to changes in sample preparation (for example the amount of conductive coating) and settings of the SEM to minimize the charge accumulation (working distance).

S4.3 EDS for elemental analysis

Energy dispersive X-ray spectroscopy (EDS) count maps are overlaid over BSD images for samples S0–S7. Figs. S6 and S7 show counts for iron and silicon characteristic X-ray spectral lines. Every sample also contains smaller contributions of carbon and oxygen counts.

The EDS spectra for all samples S0–S7 are presented in Fig. S8. Spectra are staggered for easier comparison. The spectra are not quantitatively significant, as they are taken from a small part of each sample surface, where local variations of elemental composition are high.

References

[1] J. I. Goldstein, D. E. Newbury, J. R. Michael, N. W. Ritchie, J. H. J. Scott, and D. C. Joy. *Scanning Electron Microscopy and X-Ray Microanalysis.* Springer New York, New York, NY, 2018.

BSD images of S0

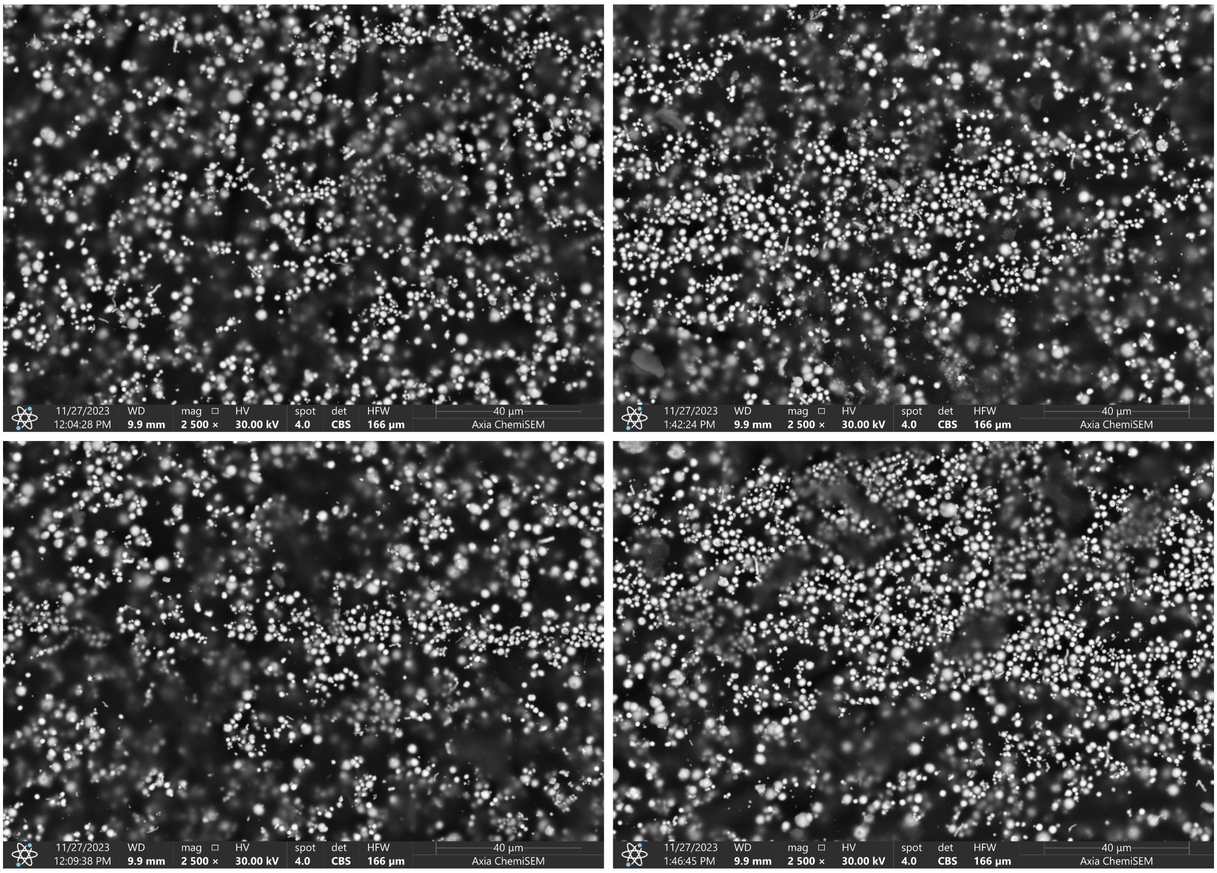

BSD images of S1

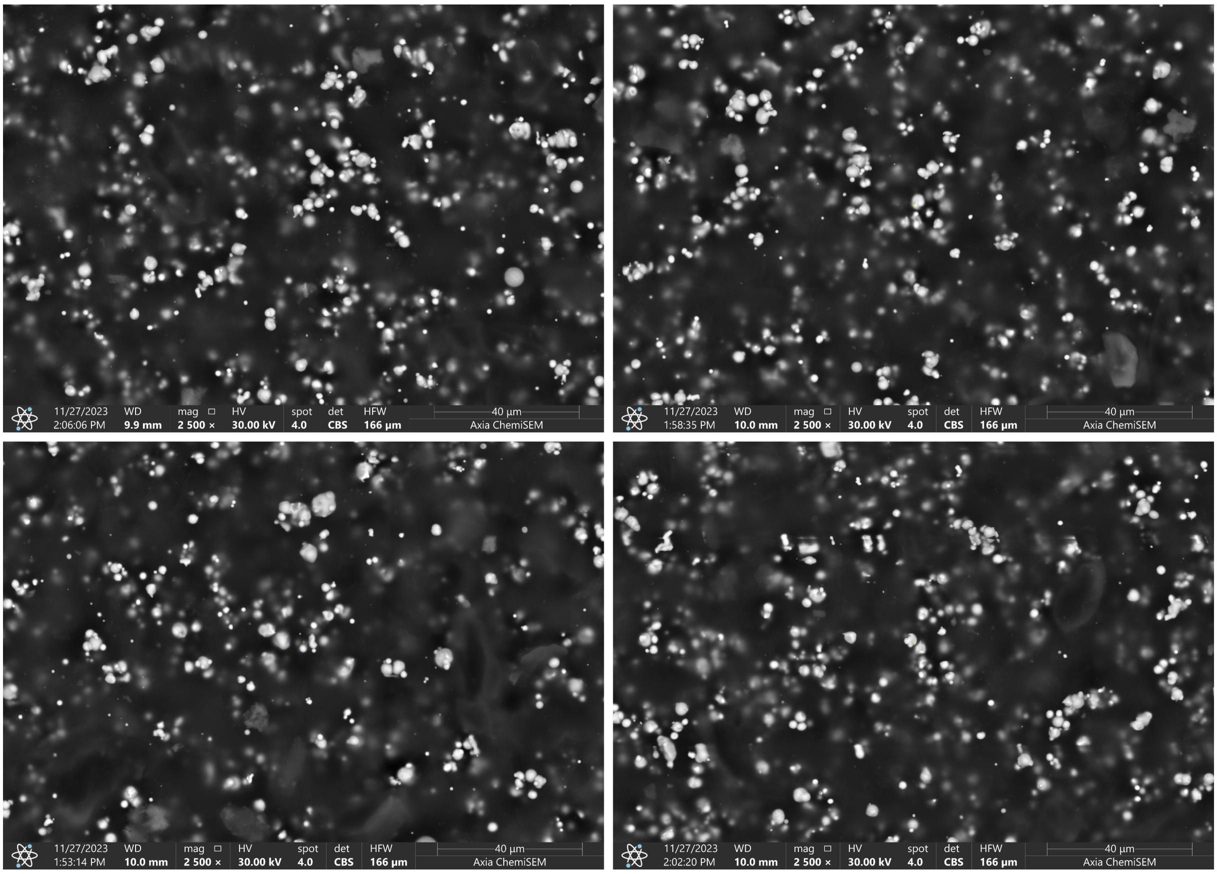

BSD images of S2

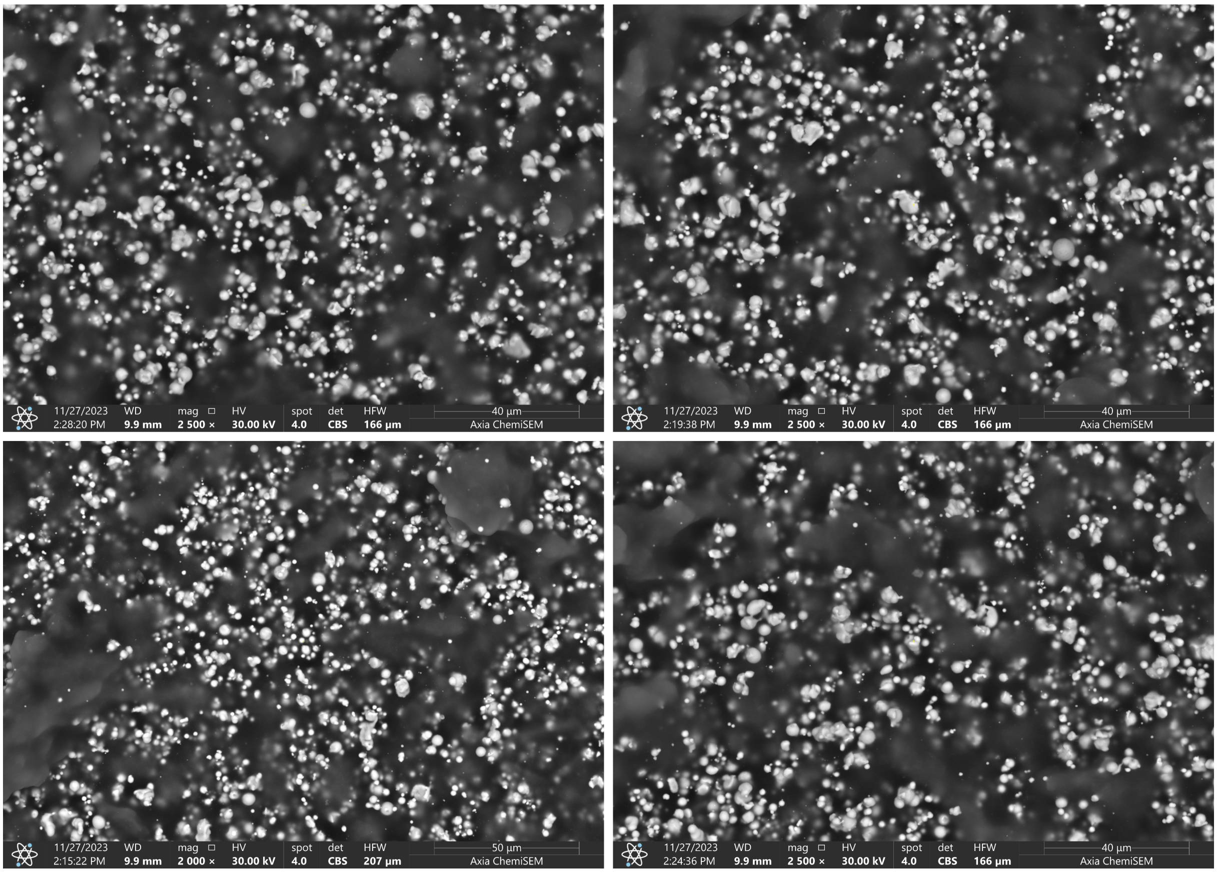

BSD images of S3

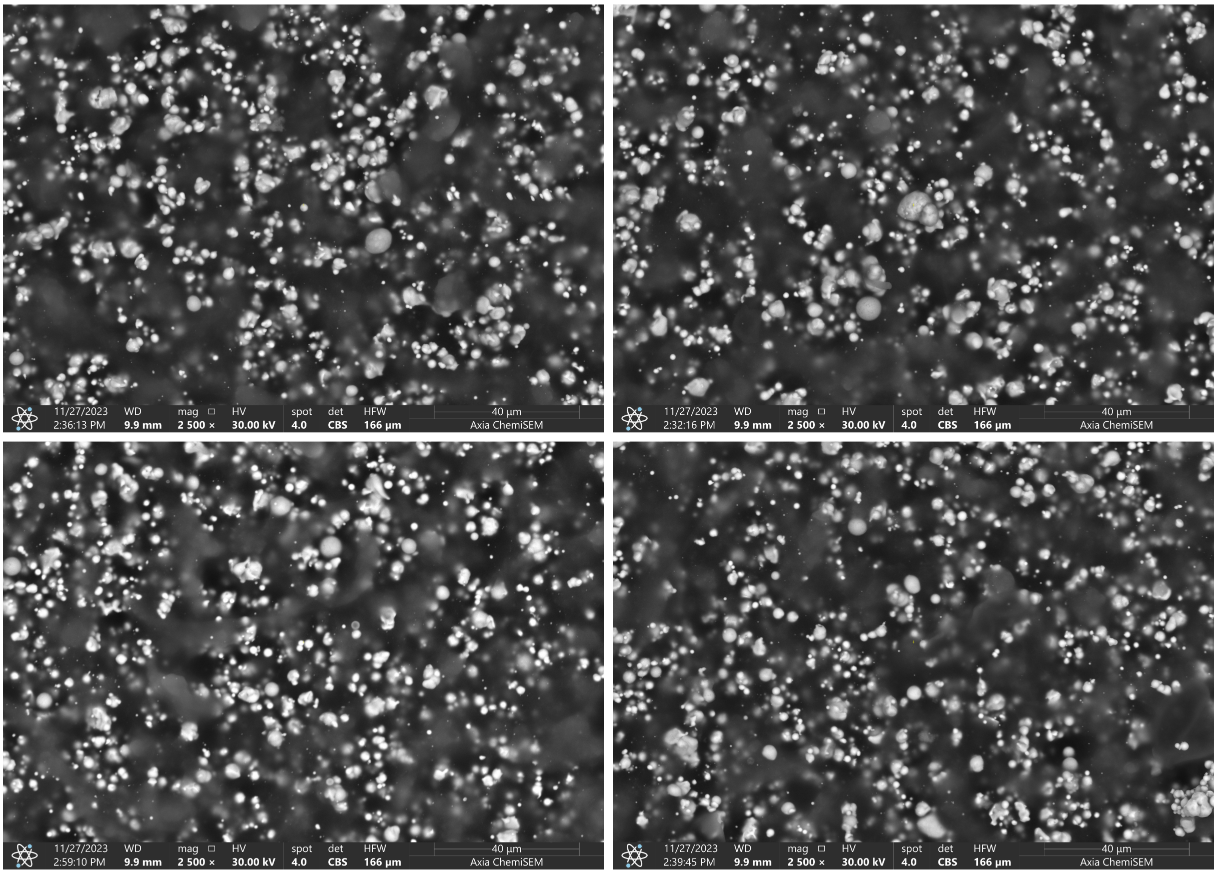

BSD images of S4

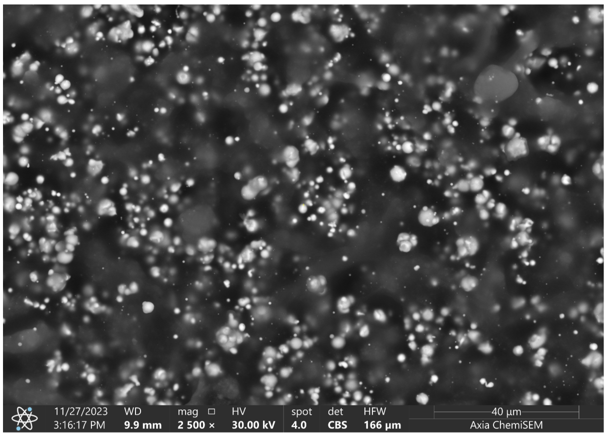

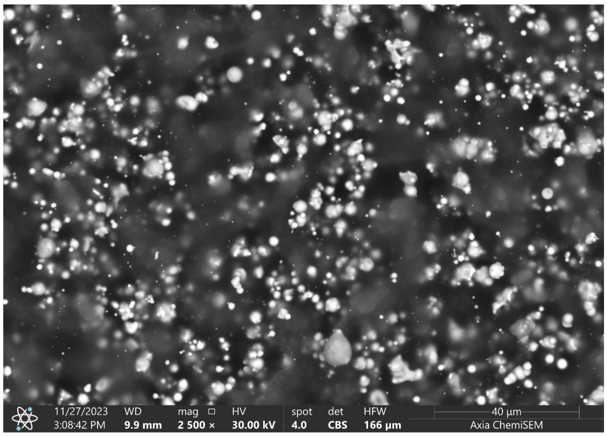

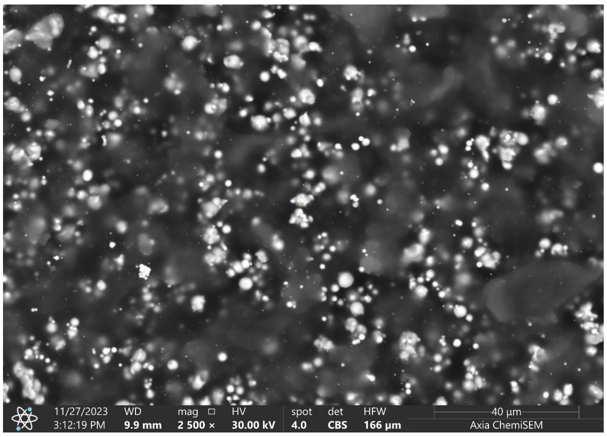

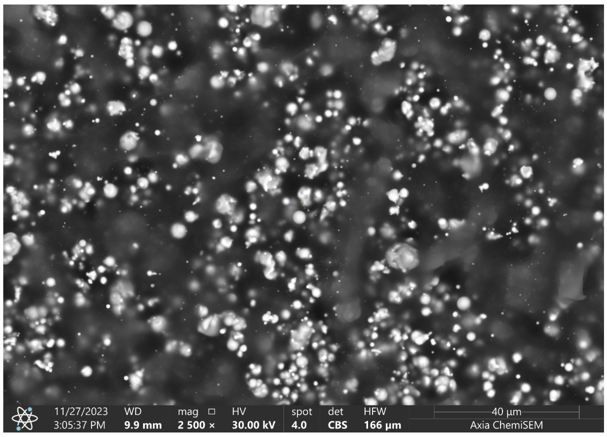

BSD images of S5

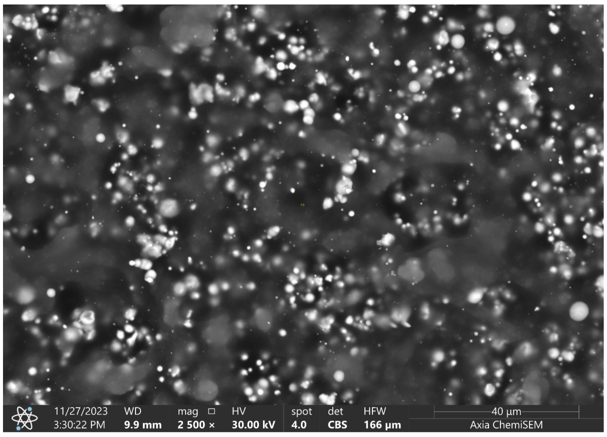

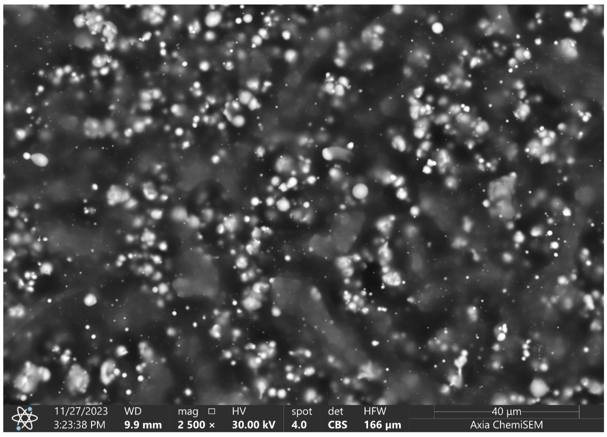

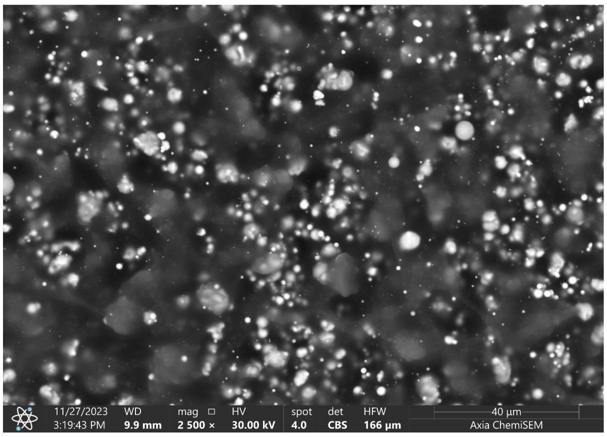

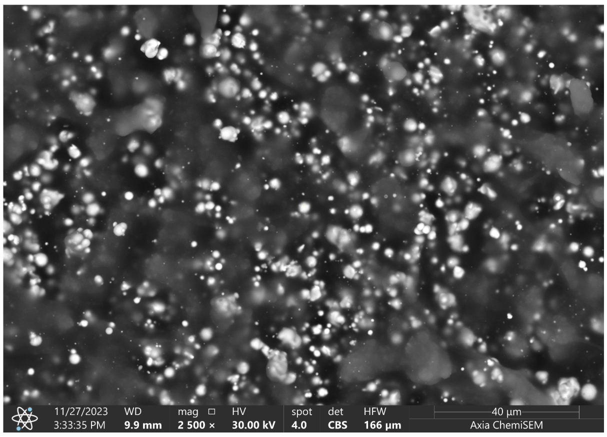

BSD images of S6

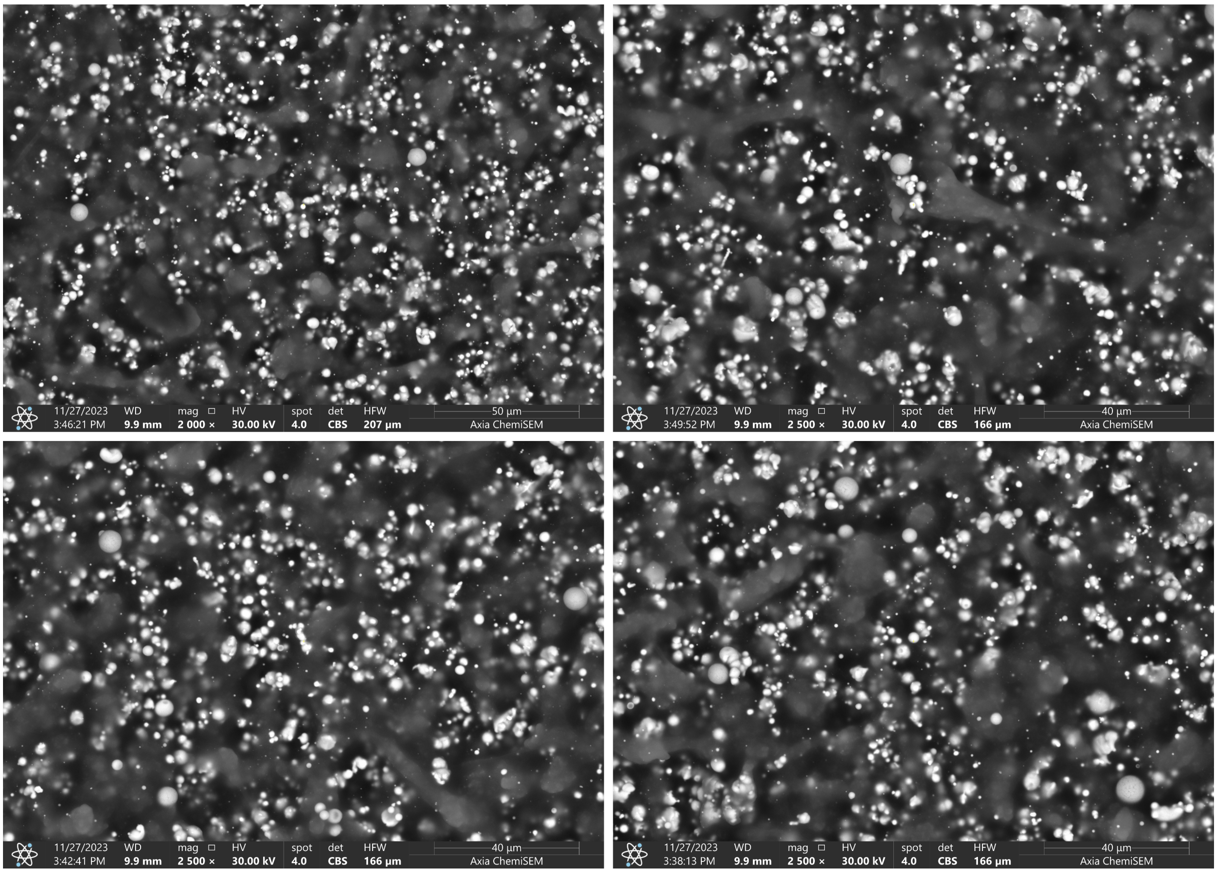

BSD images of S7

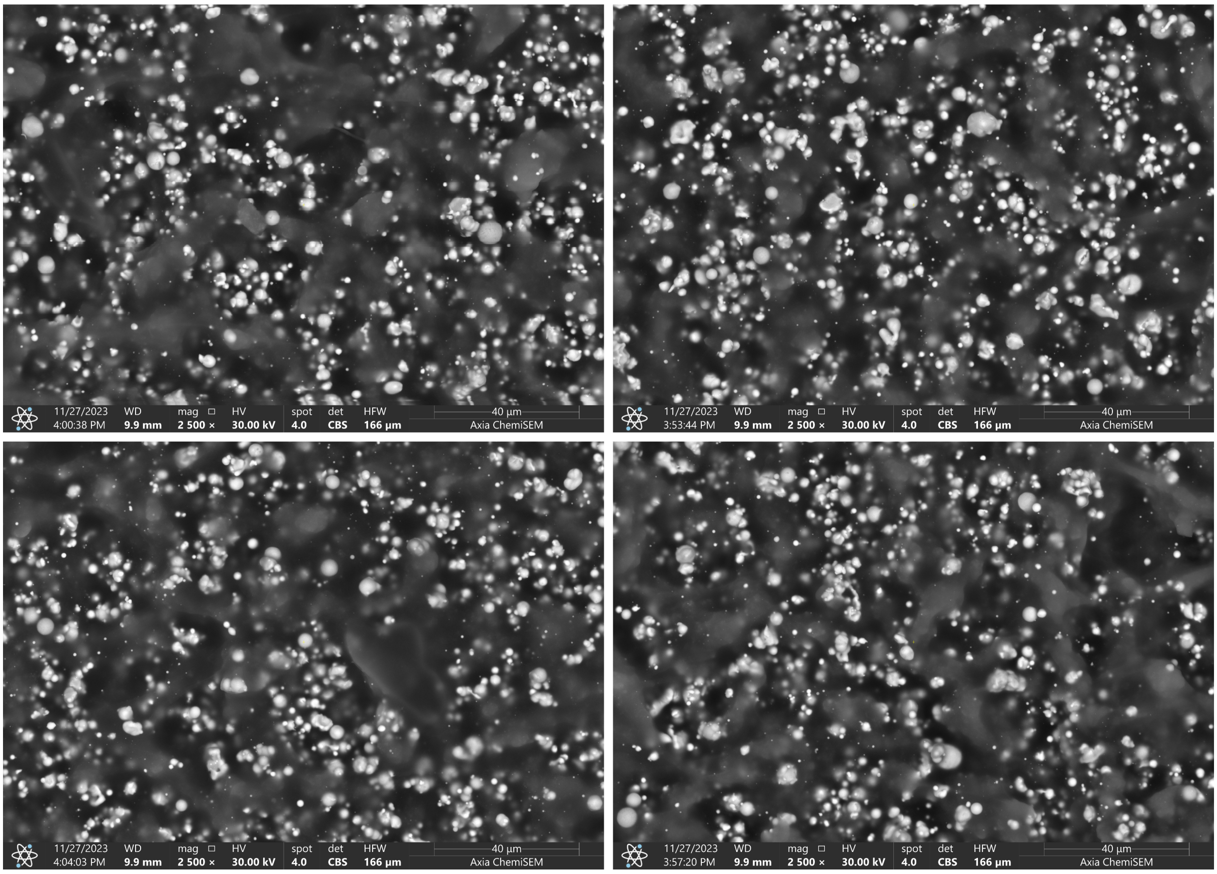

SED images of topography

S0

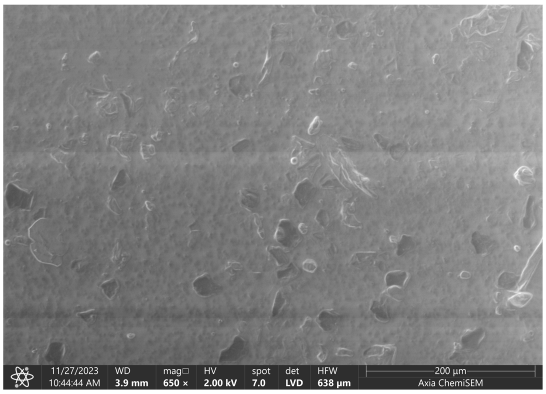

S1

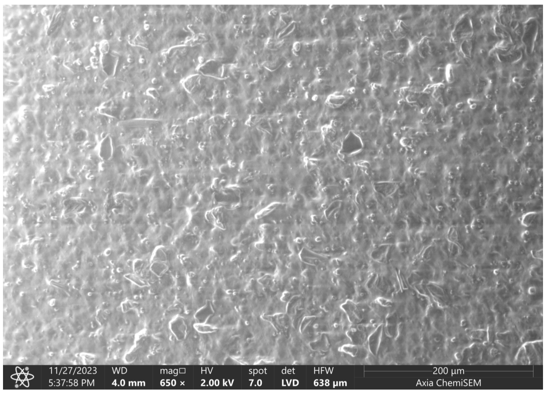

S2

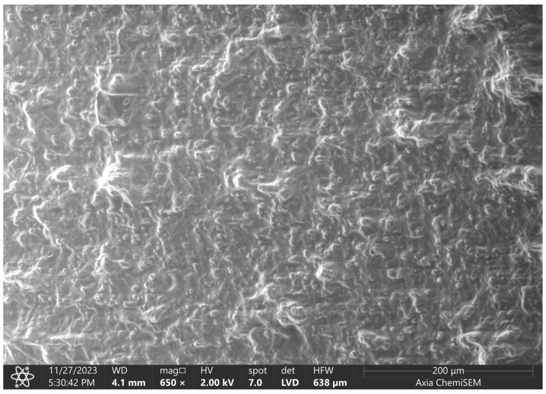

S3

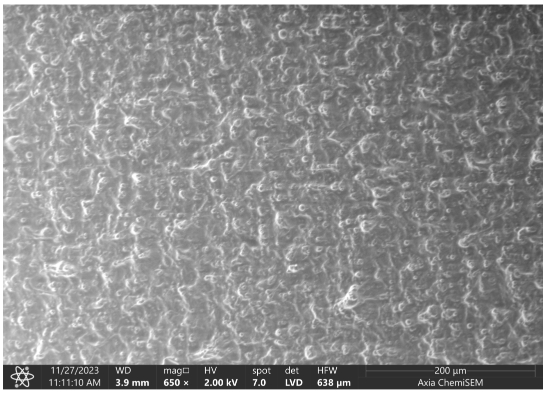

S4

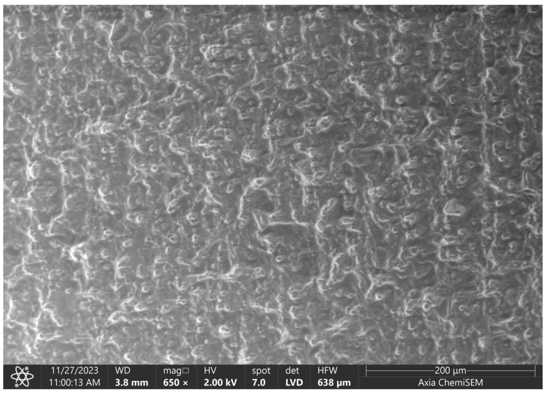

S5

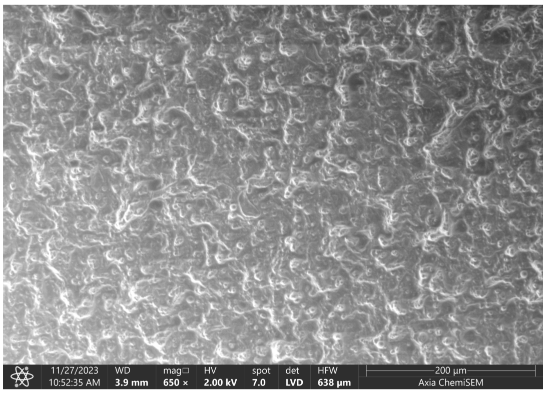

S6

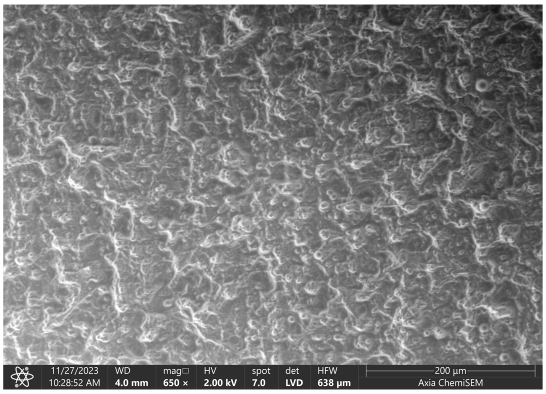

S7

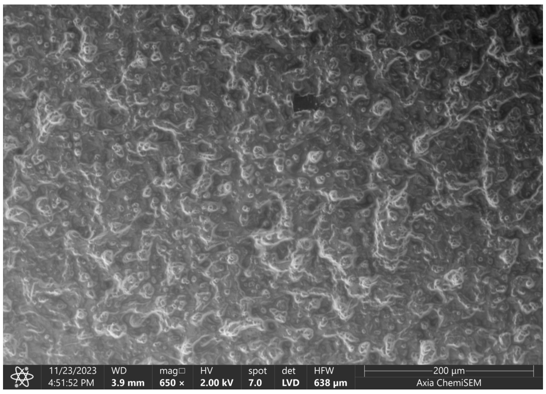

Figure S5: These raw images were preprocessed with a rolling ball background subtraction algorithm and CLAHE before computing the arithmetic mean deviation and FFT analysis described in the main text. S2 is the outlier in FFT analysis.

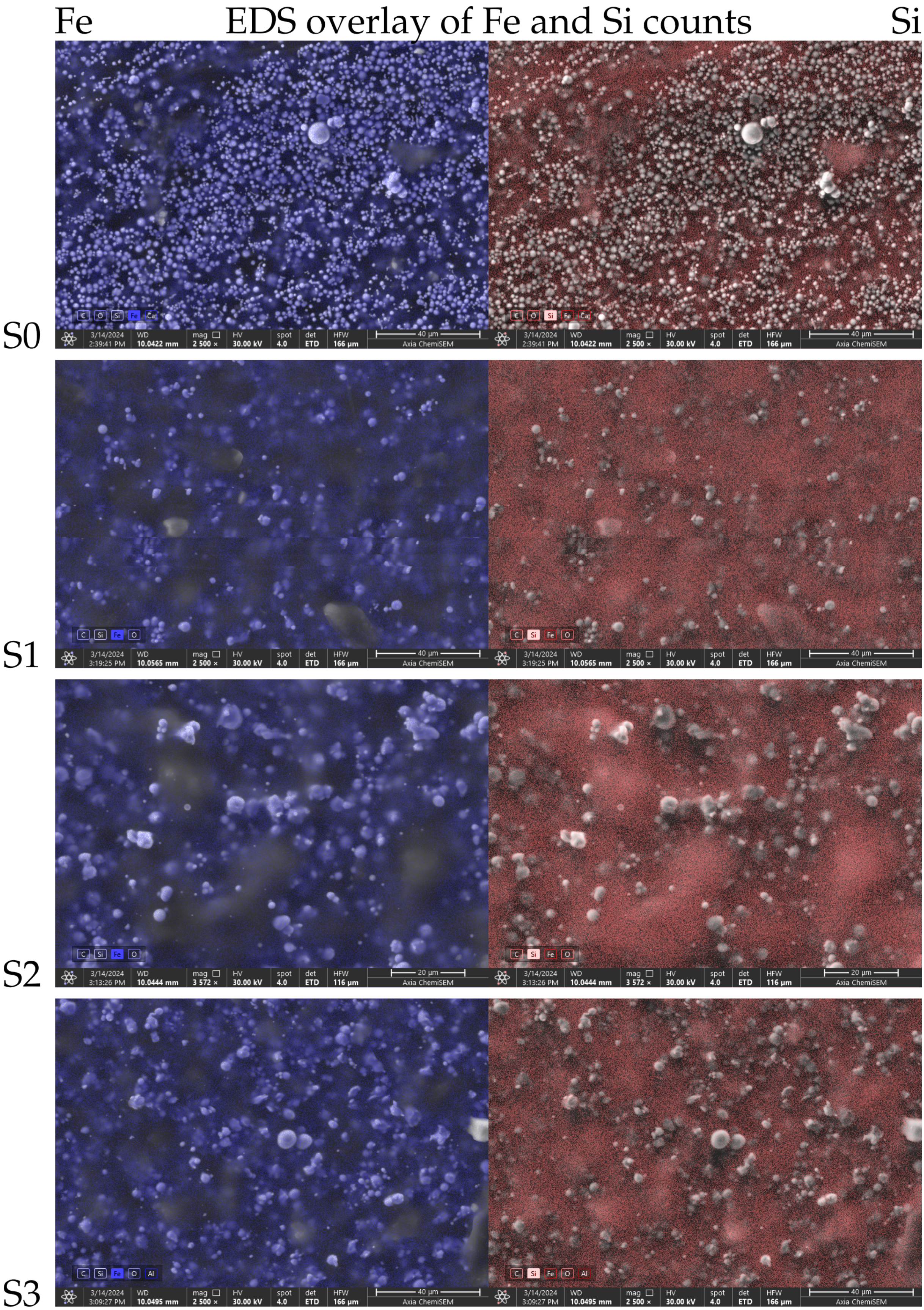

Figure S6: S2 measurement was accidentally taken at a higher magnification, however that does not affect the EDS spectrum.

Fe EDS overlay of Fe and Si counts Si

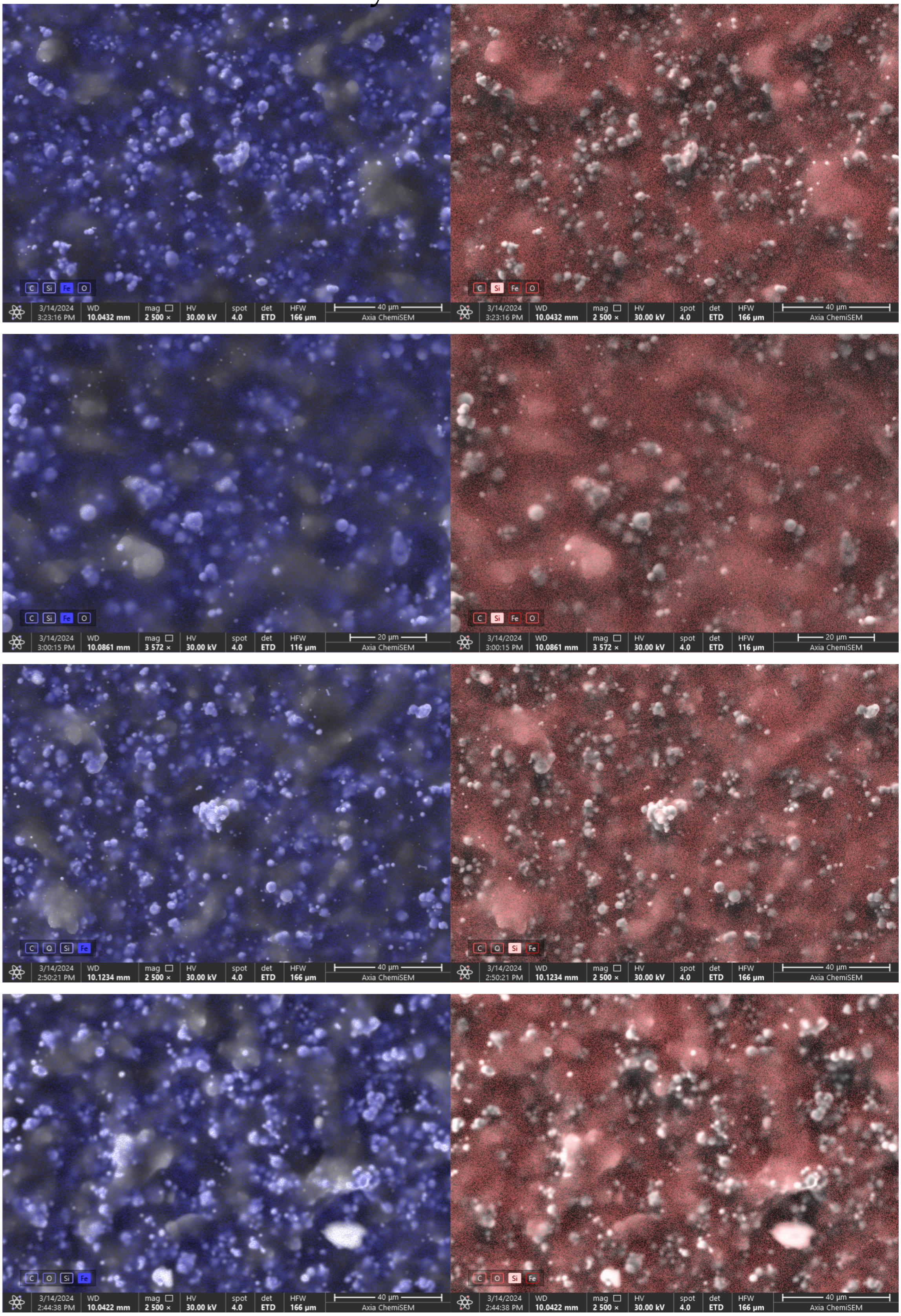

S4

S5

S6

S7

Figure S7: S5 measurement was accidentally taken at a higher magnification, however that does not affect the EDS spectrum.

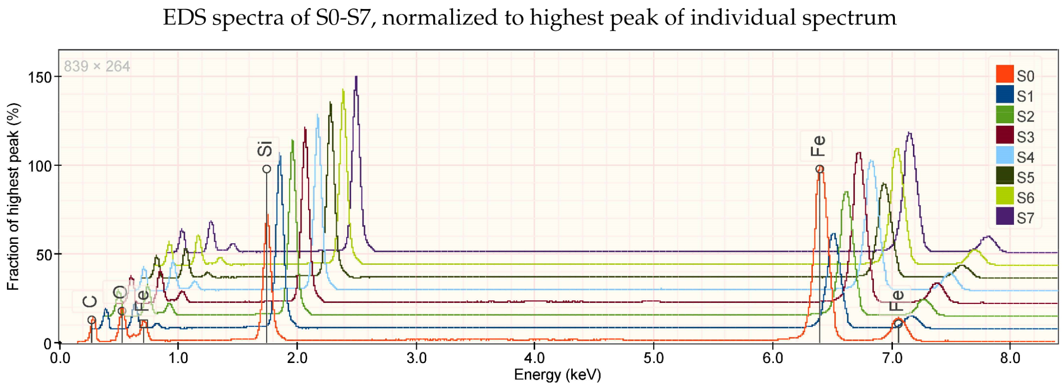

Figure S8: EDS spectra for S0–S7, taken at one arbitrary part of each sample surface, seen on Figs. S6 and S7. Each spectrum is scaled to it's highest count peak.
